# Effectiveness of eye movement exercise and diaphragmatic breathing with jogging in reducing migraine symptoms: A preliminary, randomized comparison trial

**DOI:** 10.1002/brb3.2820

**Published:** 2022-12-01

**Authors:** Mohammad Dawood Rahimi, Pouriya Hassani, Mohammad Taghi Kheirkhah, Javad Salehi Fadardi

**Affiliations:** ^1^ Faculty of Education and Psychology Ferdowsi University of Mashhad Mashhad Iran; ^2^ Department of Cognitive Neuroscience and Clinical Neuropsychology University of Padova Padua Italy; ^3^ Institute for Cognitive and Brain Sciences Shahid Beheshti University Tehran Iran; ^4^ School of Community and Global Health Claremont Graduate University Claremont California USA; ^5^ School of Psychology Bangor University Bangor UK

**Keywords:** diaphragmatic breathing, eye movement exercise, jogging, migraine treatment

## Abstract

**Background:**

Migraine is a multifactorial headache disorder. Maladaptive functional networks or altered circuit‐related connectivity in the brain with migraine appear to perturb the effects of usual treatments.

**Objectives:**

In the present preliminary trial, we aim to study the effectiveness of performing pieces of body–mind, cognitive, or network reconstruction‐based training (i.e., eye movement exercise plus jogging; EME+J and diaphragmatic breathing plus jogging; DB+J) in decreasing migraine symptoms.

**Methods:**

We used a three‐arm, triple‐blind, non‐inferiority randomized comparison design with pre‐test, post‐test, and follow‐up measurements to assess the effectiveness of EME+J and DB+J in the brain with migraine. Participants were randomly assigned to one of the study groups to perform either 12 consecutive weeks of EME+J (*n* = 22), DB+J (*n* = 19), or receiving, treatment as usual, TAU (*n* = 22).

**Results:**

The primary outcome statistical analysis through a linear mixed model showed a significant decrease in the frequency (*p* = .0001), duration (*p* = .003), and intensity (*p* = .007) of migraine attacks among the interventions and measurement times. The pairwise comparisons of simple effects showed that EME+J and DB+J effectively reduced migraine symptoms at the post‐test and follow‐up (*p* < .05). Cochran's tests showed that interventions decreased the number of menses‐related migraine attacks. EME+J and DB+J effectively decreased over‐the‐counter (OTC) drug use, refreshed wake‐up mode, and improved sleep and water drinking patterns. These are the secondary outcomes that Cochran's tests showed in the interventional groups after the interventions and at 12 months of follow‐up.

**Conclusion:**

EME+J or DB+J can be an effective and safe method with no adverse effects to decrease the symptoms of migraine attacks. Moreover, a reduction in the frequency of menstrual cycle‐related attacks, OTC drug use, and improved quality of sleep and drinking water were the secondary outcomes of the post‐test and a 12‐month follow‐up.

## Graphical abstract

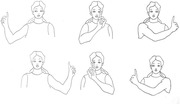

## SIGNIFICANT OUTCOMES

Performing 12 weeks of eye movement exercises with jogging or diaphragmatic breathing with jogging:

1. can significantly reduce the measured aspects of migraine pain characteristics.
2. offers a safe and convenient intervention.
3. produces no short‐term or long‐term undesirable side effects.

## 1 INTRODUCTION

Alterations in response to daily life stressors and neuronal excitability hint at a common disorder such as migraine (Chan et al., 2019; Cordero et al., 2012; Gazerani, 2021; Goadsby, 2006; Park et al., 2016; Scheffer et al., 2013). Examples of such stressors could be maladaptive stress responses in both peripheral and central nervous systems (Ashina, 2020). The symptoms of migraine attacks depend on the activation and sensitization of first order (i.e., cranial neurons) and sensitization and altered habituation of second order (i.e., cortical neurons). The quantity, duration, and presence of other elements show activation and sensitization or stress response activities in the brain with the symptoms of migraine. For example, pituitary adenylate cyclase‐activating or calcitonin gene‐related peptides can affect genetic modulation, modulation of cell communication (e.g., transcription factors), and cerebral blood flow or vascular tone alteration (e.g., vasodilation) (Ashina, 2020). For instance, eye–brain electrochemical transduction and retinal nerve fiber alterations, structural and functional abnormalities in the endothelial progenitor cells, interlinks between mitochondrial energy production, reactive oxygen species availability (e.g., nitric oxide—NO), and ion homeostasis (e.g., calcium influx) seem to play initiating roles in a brain with migraine (Ashina, 2020; DeBuc et al., 2020; Lee et al., 2008; Polak et al., 2003; Zhang et al., 2001). Therefore, regulation and availability of signaling biomolecules (e.g., NO, and brain‐derived neurotrophic factor—BDNF) and regulation of signaling pathways (e.g., cAMP‐response element‐binding protein—CREB) are essential during health and illness, including the symptoms of migraine attacks, as stressful insults to the brain (González‐Rodríguez et al., 2019; Khosravi et al., 2019).

Although nonsteroidal anti‐inflammatory drugs (NSAIDs), antidepressants, triptans, gepants, and anti‐epileptic drugs are the frequently used medications for the symptoms of migraine attacks, most of these medications have low therapeutic gain, low resolution of symptoms, and undesirable side effects (Picón‐Pagès et al., 2019; Tfelt‐Hansen, 2021). For example, in the presence of triptans or morphine, NO boosts the activation of trigeminal pathways or increases the risk of medication overuse headache (MOH) (Tepper, 2012). Nevertheless, the chronic administration or high doses of antidepressants suppress the expression of the signaling biomolecule genes (Al‐Hasani & Bruchas, 2011; Lüscher & Malenka, 2011; Xu et al., 2003). Moreover, manipulating BDNF gene expression may contribute to developing other disorders or diseases, including anxiety, depression, refraction, and chronification of migraine (Androulakis et al., 2017). Considering the complications, developing novel, safe, multidimensional, and non‐pharmacological interventions offer a promising possibility in managing the refraction or reduction in frequency, duration, and intensity of the attacks in a brain with migraine (Fidan et al., 2006).

The effects of regular aerobic exercises or progressive relaxation techniques on pain have been documented in earlier studies (Lemmens et al., 2019; Naugle et al., 2012; Starling, 2019). The present trial assumes that regular aerobic exercises adjunct to visual exercises, exercise‐induced, nasal, or diaphragmatic breathing support brain homeostasis, improve brain function, protect the brain against various‐induced toxicities, and relieve pain experience. Inhibition of excessive presence of N‐methyl‐D‐aspartate receptors, regulation of serotonin or glutamate excitatory signaling, and promotion of BDNF gene expression are the expected positive outcomes in the present trial (Almeida et al., 2005; Andreou & Goadsby, 2009; Cotman & Berchtold, 2002; Lima et al., 2017; Manzoni et al., 1992; Marosi & Mattson, 2014; Martínez et al., 1993; Ramadan, 2003; Wang & Kriegstein, 2008; Xu, 2013; Ye & Sontheimer, 1996). That is, through regulation of local and cortical endothelium releasing factors (e.g., NO and BDNF), regular aerobic exercises, nasal breathing, and visual exercises facilitate and rearrange cortical and oscillatory feedback signaling between cranial motor nerves, brain stem, thalamus, hypothalamus, motor, and sensory cortices, all of which are closely related to a brain with migraine (Dinç et al., 2020; Dorner et al., 2003; Matteo et al., 2016; Ploughman et al., 2007; Savchenko et al., 1997; Sparks, 2002).

## 2 METHODS

### 2.1 Participants

To ensure a sufficient sample size for the present trial, we performed an a priori power analysis using G*Power (Faul et, al.). Eventually, a minimum required sample size of 63 participants was estimated to detect an effect of *f* = 0.315 at a significance level of *α* = .05 with a power of 1‐*β* = .80.

All participants in the final sample were right‐handed, Persian‐speaking female individuals (Labastida‐Ramírez et al., 2019; van den Brink & MacGregor, 2019) (mean age = 31.19; standard deviation, SD = 5.67; range of 19–40) with migraine (33.3% with aura). Participants were invited to join a scientific trial that might help with migraine treatment. The trial population consisted of 764 individuals with migraine who were admitted to two local state hospitals between September 2015 and August 2016. After the eligibility assessment (the trial flowchart below), 353 individuals did not meet the trial criteria (below), and 276 were unable to take part. Of the remaining 135 individuals, another 72 participants were randomly dropped to get to the final sample (*n* = 63) for the baseline assessment. A total of 10 participants were excluded during the follow‐up. Therefore, the final data analyses were conducted with all remaining participants in the trial. Participation in the trial was voluntary. During their enrolment, participants filled out a questionnaire on demographic information, history of migraine, socioeconomic status, and migraine type (Table 1).

**TABLE 1:** Mean and SDs for demographic information, history of migraine, and distribution of participants based on socioeconomic status and type of migraine in study groups

	Groups					
	Eye movement	Diaphragmatic breathing	Control			
Variables	Mean (SD)			F (2,60)	χ^2^	p
Age	31.15 (6.40)	31.61 (4.87)	30.73 (5.88)	0.09		.90
Weight (Kg)	61.95 (7.51)	61.39 (7.77)	62.07 (8.08)	0.03		.96
Height (cm)	163.75 (6.98)	162.50 (5.53)	162.93 (6.79)	0.18		.83
HM (yr.)	5.00 (3.06)	4.39 (2.63)	4.93 (3.36)	0.22		.80
	N (%)					
SES					1.70	.79
High	4	3	5			
Middle	14	13	8			
Low	2	2	2			
MT					2.97	.22
MwA	8	3	6			
MwoA	12	15	9			

Abbreviations. HM, history of migraine; MT, migraine type; MwA, migraine with aura; MwoA, migraine without aura in study groups.; SES, socioeconomic status.

Inclusion criteria were: (a) 18–50 years of age (Kurth et al., 2005); (b) a confirmed diagnosis of migraine based on the International Classification of Headache Disorders (ICHD) criteria (3rd edition (beta version)) ((IHS) HCCotIHS 2013); and (c) a history of migraine (range of attacks = 1–14 days for 4 weeks) over the last 12 months regardless of the migraine type (i.e., with or without aura). It should be noted that the study was started before 2018, prior to the publication of IDHD‐3 (see the study flowchart). Exclusion criteria were taking common migraine medications, including prophylactics and acute ones—the list comprised NSAIDs (i.e., ibuprofen, diclofenac, and naproxen), sedatives, selective serotonin reuptake inhibitors (i.e., fluoxetine), serotonin‐norepinephrine reuptake inhibitors (i.e., venlafaxine and duloxetine), tricyclic antidepressants (i.e., imipramine), beta‐blockers (i.e., propranolol), epileptic‐specific medications (e.g., valproate sodium and topiramate), ergot alkaloids (e.g., ergotamine), triptans, analgesics and antipyretics (e.g., paracetamol, or acetaminophen), except when they were experiencing intolerable attacks. In addition, a history of any other neurological, gut, or respiratory‐related diseases, including concomitant diagnosis of other headache disorders, especially MOH (Ferrari et al., 2015), any surgery that restricts their ability to jog, perform eye movement exercise, or breathe, or cardiovascular‐related diseases, for example, blood pressure abnormalities were excluded. Individuals in the interventional groups agreed not to take pain medications, including prophylactics or other interventions, for at least 3 months before and during the present trial. If a participant was taking medication, they were given 45 days to quit the medication, and 15 days after dropping the medication, they entered one of the research groups—because the effects of prophylactic or other drugs continue for a while. However, if they were experiencing severe pain, they could use limited medication prescribed by their physician. The trial flowchart shows the flow of the present trial from 2016 to 2019.

After their diagnosis was confirmed by two consultant neurologists at a local state hospital, based on ICHD criteria (3rd edition [beta version]) ((IHS) HCCotIHS 2013), participants had to meet the inclusion criteria and be flexible with the trial's terms. The sample was assessed over 46 weeks between August 2016 and July 2017. We used a paper‐based baseline headache diary to screen the sample for frequency, duration, and intensity of the attacks in the last 3 months (Diener et al., 2020; Niere & Jerak, 2004). Next, using the random digit table, participants were randomly (1:1:1) assigned to one of the interventional groups, that is, eye movement exercise (*n* = 22) or diaphragmatic breathing (*n* = 19) or to the control group (*n* = 22). All the data were recorded by three research assistants who were blind to the trial design. One co‐author conducted the data analysis and was blind to the trial design and its hypotheses.

#### 2.1.1 Ethics approval and informed consent

The present trial was performed in line with the Declaration of Helsinki and was approved by the ethics committee of Mashhad University of Medical Sciences (Ir.mums.fm.rec.1396.362, 22/06/2017). The recruited participants in the present trial were from a project on migraine, from which a randomized controlled trial has already been published (Rahimi et al., 2020). Written informed consent was obtained from the participants.

All the methods were performed under the Guidelines of the International Headache Society (IHS) for controlled trials of preventive treatments of migraine in adults (Diener et al., 2020). The participants were told that their participation was voluntary and that they could withdraw from the experiment at their will. They were told that they were part of a trial on migraine.

### 2.1 Materials

#### 2.2.1 Headache diary

We used a paper‐based, baseline headache diary (Niere & Jerak, 2004; Tassorelli et al., 2018) to record the frequency, duration, and intensity of the pain per migraine attack during the past 4 weeks—over 3 months. The frequency could get any of the following forms: (a) experiencing one headache day per 4 weeks; (b) one headache day in 2 weeks; (c) two or three headache days per week; or (d) more than three headache days per week. The duration included 4, 4–24, or 24–72 h per attack. The intensity was moderate (1–3), severe (4–7), or worse case (8–10) per migraine attack during the past 4 weeks—over 3 months. The measure has desirable reliability and validity indices (Niere & Jerak, 2004; Tassorelli et al., 2018).

#### 2.2.2 Secondary outcome measures

Prior to the trial, a research assistant recorded information from the participants on (a) menstrual cycle; (b) over‐the‐counter (OTC) drug use; (c) sleep regimens (sleep and wake‐up patterns), sleeping hours, wake‐up mode (Panda, 2020); (d) drinking water; and (e) exercise intolerance. The menstrual cycle involves the presence or absence of effects on headache characteristics before, during, or after a menstrual period. Sleep patterns involve sleeping on time (between 9:00 p.m. and 11:00 p.m.) or late at night (after 11:30 p.m.). Sleeping hours included sleeping less than or more than 7 h per night (between 10:00 p.m. and 7:00 a.m.) (Bertisch et al., 2018; Finan et al., 2013). In addition, drinking water was also recorded for the participants (Barraj et al., 2009). However, any incidents of muscle cramps, fatigue, chronic dizziness, and vomiting related to exercise intolerance were recorded. The data were recorded every other day during the experiment and every week during the 12‐month follow‐up. The rationale for recording and analyzing this data was to assess whether the interventions influenced such variables besides migraine pain characteristics.

### 2.2 Procedures

In the present non‐inferiority randomized comparison trial, the possible concealment (i.e., keeping an optimum strategy (Karanicolas et al., 2010)) was preserved to prevent the impact of individuals' expectations or biases on their responses or behaviors. Therefore, participants and research assistants responsible for interventions and data collection were blind to group allocation. Moreover, the intervention time for all interventional groups was equal (Piaggio et al., 2006; Shah, 2011).

We used a baseline headache diary over 3 months to measure pain duration, frequency, and intensity. The trial measures were administered at the baseline, post‐test, and 12‐month follow‐up. Moreover, weekly telephone follow‐ups were made to enquire about the participants’ experiences of pain, use of prescribed or non‐prescribed medication, menstrual cycle, sleeping pattern, drinking water, or other related behaviors for both interventional and control groups. The telephone calls also helped minimize the participants’ dropouts.

In case of experiencing the symptoms of migraine attacks or complications that could affect their regular daily lives, the related research assistant reassured the participants that they could have control visits with their physician during the trial. However, none of the participants in the interventional groups reported referring to their physician or resuming the use of migraine prescribed or non‐prescribed medication at any of the interventional groups' phone calls or follow‐up assessments. Therefore, the interventional groups had no data on prescribed or non‐prescribed medication relapse. The same experimenters administered all trial measures at the baseline, post‐test, and follow‐up assessments.

#### 2.3.1 Randomization

Because the present trial was based on a triple‐blind design, clinicians, experimenters, and research assistants (i.e., as many individuals as possible) who were collaborating with the individuals were unaware of the nature of the trial design and procedures. Participants were informed only about what they were instructed to do but not about their group allocation, other participants in the trial, or the trial design and goals.

To reduce the risk of prediction for a sample size of *n* > 50, we used telephone (secure) allocation of participants by an independent person in line with instructions for a simple random method. Before continuing to the next stage, the inclusion and exclusion criteria of the present trial were watched and checked. For example, if a participant was on prescribed or non‐prescribed medication, they were asked to stop their medication taperingly (i.e., over 6 weeks). The effects of prescribed or non‐prescribed medications are still for a while. To reduce the risk of missing the participants, they were at once included in the experiment groups. Nonetheless, for the control group, there was no regimen (i.e., they were following their regular medication intake under the consultant of their physicians) for their medication intake before, during the intervention, or over the follow‐up. Moreover, individuals were asked and screened for any types of smoking, but there were no reports of having a smoking habit.

One research assistant was randomly assigned to each group to execute the participants’ enrollment and intervention assignments during the trial. Using a paper‐based diary, the research assistants were instructed and scheduled to check and record medications, menstrual cycles, sleeping, drinking water, and exercise intolerance behaviors in the interventional and control groups.

#### 2.3.2 Interventions

There were two interventional groups. The first interventional group performed eye movement exercise and the second interventional group performed diaphragmatic breathing instruction. Likewise, there was an active control group. In the control group, the common pharmaceutical intervention was presented.

**FIGURE 1:**
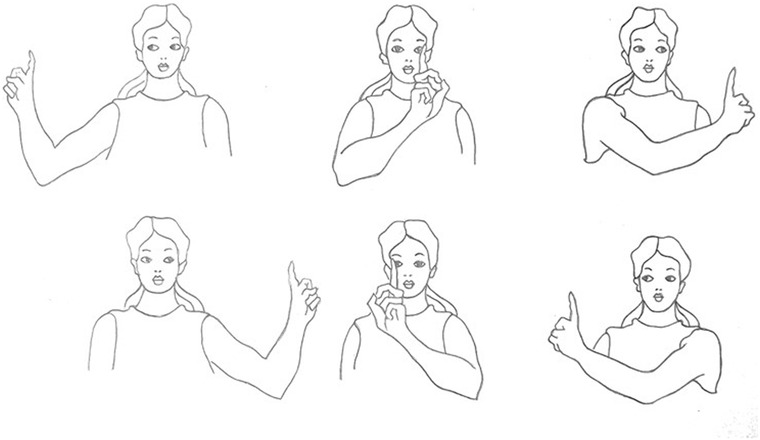
Shows participants' eye movement exercise for interventional groups: The first row shows vertical head‐guided eye movement exercise; the second row shows horizontal head‐guided eye movement exercise.

**FIGURE 2:**
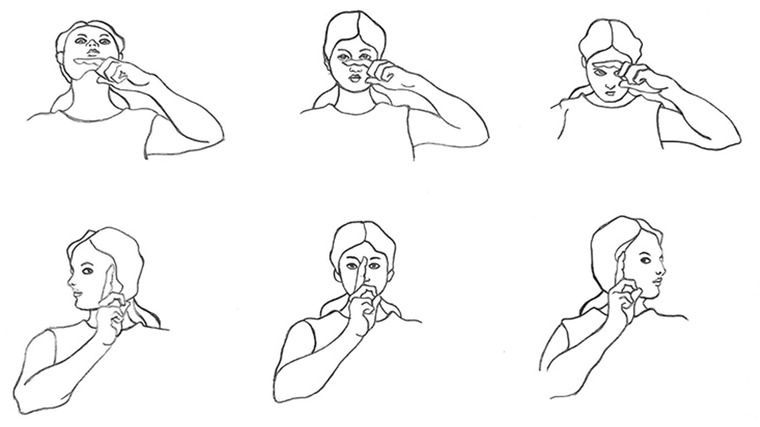
Shows participants' eye movement exercise for interventional groups: The first row shows vertical head‐guided eye movement exercise; the second row shows horizontal head‐guided eye movement exercise.

The eye movement exercise included horizontal (dextroversion/levoversion), and vertical (sursumduction/deorsumduction) conjugate eye movements (Jain, 2019). Participants performed a 5 min eye movement exercise 30 min before having their breakfast every morning and 30 min before going to bed at night for 12 weeks. The 5 min bedtime phase was started by 15‐round movements of the right‐hand index finger, followed by 15‐round left‐hand index finger movements in the horizontal visual field, which was followed by the same procedure in the vertical visual field with a front‐forward, fixed head with the eyes following the finger in a constantly controlled manner (Figure 1). The 5 min morning phase was initiated, sustained, and terminated with the same procedure, but instead of moving the index finger in the visual field, the head was moved horizontally (30 rounds laterally) and then vertically (30 rounds up and down) with fixed eyes on an index finger, which was fixed in the visual field (Figure 2) (Leigh & Zee, 2015). Figures 1 and 2 show participants’ hands and head‐guided eye movement exercises in the interventional groups.

The diaphragmatic breathing practice required a deep nasal inhalation, a 2 s repose, and a long, deep oral exhalation three times a day around 7:00 a.m., 2:00 p.m., and 9:00 p.m. for 5 min for 12 consecutive weeks. Participants were persuaded to focus on their breathing while inhaling and exhaling (Adler et al., 2014; Guyenet Patrice & Bayliss Douglas, 2015).

**FIGURE 3:**
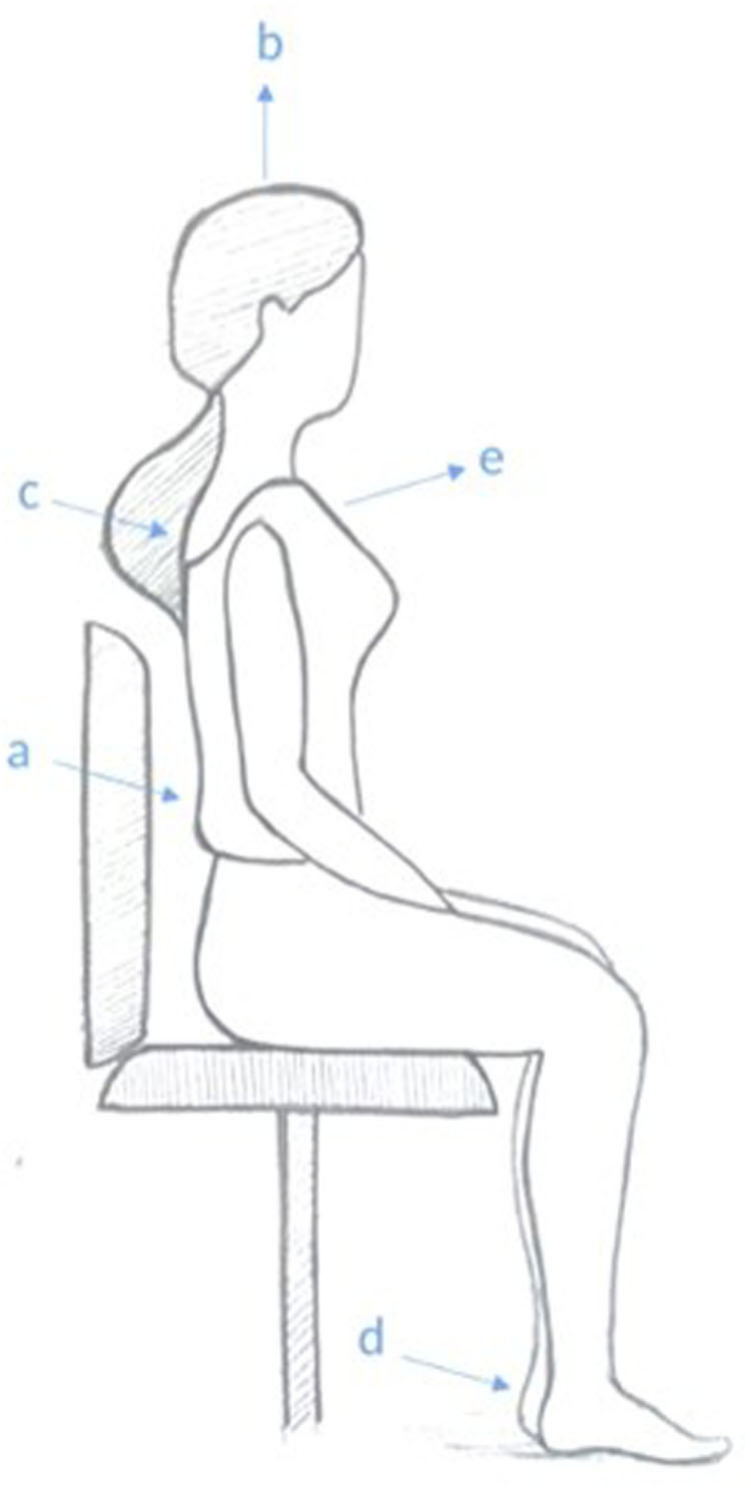
Participants’ posture during eye movement exercise or diaphragmatic breathing: a = upright back; b = fixed, leveled, and high‐up head; c = rolled backed shoulders; d = planted feet on the ground at a 90‐degree angle to the extension of shoulders; e = slouched forward breast

During the eye movement exercise and diaphragmatic breathing practice, participants were asked to sit upright and plant their feet on the ground at a 90‐degree angle to match the extension of their shoulders. Moreover, they were instructed to fix their heads horizontally, leveling with their bodies. They were also asked to arch their backs completely and slouch forward. The position was followed by rolling shoulders back and dropping them down. The preparation phase ended with a deep breath and relaxation (Figure 3) (Jo, 2019).

The active control group received the medical intervention, which consisted of commonly prescribed medication to help with the migraine symptoms. Because there were a variety of prescribed prophylactic medications (e.g., propranolol, imipramine) and their effects on the migraine symptoms were not a goal for the present trial, it was not necessary to keep a record of the type of medication that participants were using throughout the trial. Therefore, like the interventional groups, they were checked regularly to reduce the attrition rate and completed all the trial measures at the baseline, post‐test, and follow‐up assessments.

After the randomization and group allocation, two research assistants who were blind to the aims and nature of the trial were instructed to train the participants on how to perform eye movement exercises and diaphragmatic breathing in two face‐to‐face sessions (each session = 30 min). Nonetheless, jogging was instructed in a separate and face‐to‐face session (30 min) by another research assistant.

All members in the interventional groups had to go jogging during the intervention they received. Jogging included circling the outer ring of a local public park for 3.2 km (≈ 25 min). During jogging, the instruction was that they followed a breathing rhythm that needed two relaxed, deep nasal inhalations, a repose, and two deep oral exhalations. The jogging was exercised every other day (between 5:00 and 7:00 p.m.) for 12 weeks.

### 2.3 Compliance with intervention

Based on the recorded data, which included 63 participants in the trial, and concluded 22 participants in the eye movement exercise, 19 participants in diaphragmatic breathing, and 22 participants in the treatment as usual (TAU) group, the overall compliance was calculated as follows: 63/63 × 100 = *x*% for the whole trial, 22/22 × 100 = *x*% compliance for eye movement exercise, and TAU groups and 19/19 × 100 = *x*% compliance for diaphragmatic breathing group (van der Horst et al., 2015).

Moreover, the trial included 36 sessions of jogging and 72 sessions of eye movement exercise or 72 sessions of diaphragmatic breathing for each trial group; compliance with the intervention was calculated as follows: 36/36 × 100 = *x*% compliance or 72/72 × 100 = *x*% compliance for each trial group (Jo, 2019). During 36 sessions of jogging and 72 sessions of eye movement exercise or diaphragmatic breathing, each group of participants could skip sessions for a maximum of three (with minimum compliance of 91.6%) or six (with minimum compliance of 95.8%) sessions (van der Horst et al., 2015). Because there were no reports beyond the minimum compliance with procedures, further measurement was considered unnecessary in the data analyses.

### 2.4 Data analysis

A linear mixed model (LMM) was used to decide the between‐subjects (interventions), within‐subjects (times), and interactive effects of these two factors on migraine symptoms. To ensure that there were no missing data, a priori data analysis was performed for the interventional and control groups. Homogeneity, linearity, and normality of variance‐covariance matrices were calculated, and a *p*‐value ≥ .05 was set for all analyses. Likewise, the models’ fitness tests did not violate underlying assumptions.

We considered pain characteristics (i.e., frequency, duration, and intensity of the attacks) as response variables, interventions (three levels) as the between‐subjects factor, and the time (pre‐test, post‐test, and follow‐up) as the within‐subjects factor. Moreover, as secondary analyses, Cochran's tests were calculated to measure the effects of the two types of interventions on the menstrual cycle, OTC, sleep, and drinking water—that is, behavioral outcomes from baseline to post‐test and the 12‐month follow‐up.

## 3 RESULTS

Table 2 shows means and standard deviations for frequency, duration of attacks, and pain intensity in each trial group at baseline, post‐test, and the 12‐month follow‐up assessments.

**TABLE 2:** Mean and SDs for frequency, duration, and intensity of migraine pain in each study group across three assessment points

Groups; Mean (SD)	Frequency			Duration			Intensity		
	Pre‐test	Post‐test	Follow‐up	Pre‐test	Post‐test	Follow‐up	Pre‐test	Post‐test	Follow‐up
EME	9.15 (2.68)	3.61 (1.24)	3.44 (1.79)	8.10 (3.29)	2.89 (0.67)	3.11 (1.41)	6.50 (1.67)	2.94 (.72)	3.50 (1.29)
DB	8.22 (4.00)	3.61 (1.46)	4.70 (1.21)	6.50 (2.77)	2.80 (1.36)	2.95 (0.99)	6.11 (1.32)	2.20 (0.83)	3.20 (0.95)
Control	8.80 (2.48)	6.47 (2.53)	7.33 (2.96)	8.07 (3.97)	5.93 (3.82)	5.80 (3.07)	7.07 (1.79)	4.80 (1.56)	4.20 (1.26)

*Notes*: Frequency: headache days for four weeks—over 3 months; Duration: 4–72 h per attack; Intensity: 1–10 from a visual analog scale (VAS).

Abbreviations: EME, eye movement exercise; DB, diaphragmatic breathing.

### 3.1 Primary data analyses

#### 3.1.1 Effects on frequency

**FIGURE 4:**
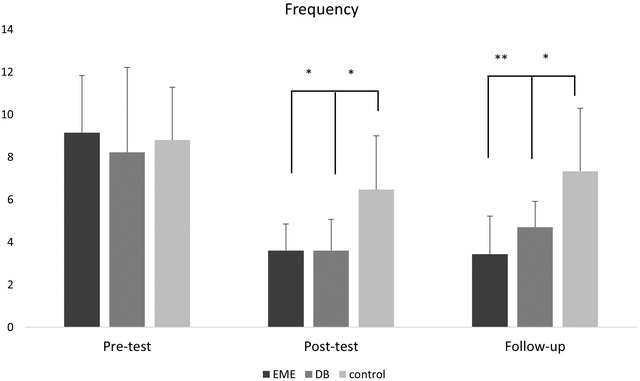
The frequency could get any of the following forms: (a) experiencing one headache day per 4 weeks; (b) one headache day in 2 weeks; (c) two or three headache days per week; or (d) more than three headache days per week during the past 4 weeks—over 3 months. EME, eye movement exercise; DB, diaphragmatic breathing, and control across three assessment points—that is, pre‐test, post‐test, and follow‐up. Note. * = *p* < .05; ** = *p* < .001

The LMM was run for the frequency of headaches. The results showed that the interaction effect among fixed factors was significant (*F* (4, 100) = 6.703; *p* = .000), which shows that both between‐groups and within‐groups factors affected the response variable, and none of them can be excluded from the model. Regarding the main effects, a significant difference in frequency was observed between the groups (interventions; *F* (2, 50) = 6.705; *p* = .003), meaning that interventions affected the frequency of migraine attacks. Likewise, a significant difference was observed within the groups (time; *F* (2, 50) = 94.848; *p* = .0001), which means that migraine frequency alteration depends on the time. A pairwise comparison of alpha‐adjusted simple effects of migraine frequency within groups is shown in Table 3. Besides, the pairwise comparison of the simple effects of headache frequency among the groups is shown in Figure 4.

**TABLE 3:** Pairwise comparisons over the time points—By groups and response variables

Groups	Responses	Time I	Time j	Mean difference	SE	sig
EME	Frequency	Pre‐test	Post‐test	4.61	0.78	.00
		Post‐test	Follow‐up	0.16	0.42	.99
	Duration	Pre‐test	Post‐test	3.61	0.66	.00
		Post‐test	Follow‐up	−0.22	0.28	.99
	Intensity	Pre‐test	Post‐test	3.16	0.35	.00
		Post‐test	Follow‐up	−0.55	0.30	.25
DB	Frequency	Pre‐test	Post‐test	5.75	0.64	.00
		Post‐test	Follow‐up	−1.30	0.36	.01
	Duration	Pre‐test	Post‐test	5.30	0.55	.00
		Post‐test	Follow‐up	−0.15	0.22	.99
	Intensity	Pre‐test	Post‐test	4.30	0.41	.00
		Post‐test	Follow‐up	−1.00	0.31	.01
control	Frequency	Pre‐test	Post‐test	2.33	0.39	.00
		Post‐test	Follow‐up	−0.86	0.32	.05
	Duration	Pre‐test	Post‐test	2.46	0.29	.00
		Post‐test	Follow‐up	0.13	0.73	.99
	Intensity	Pre‐test	Post‐test	2.33	0.23	.00
		Post‐test	Follow‐up	−0.60	0.46	.65

#### 3.1.2 Effects on duration

**FIGURE 5:**
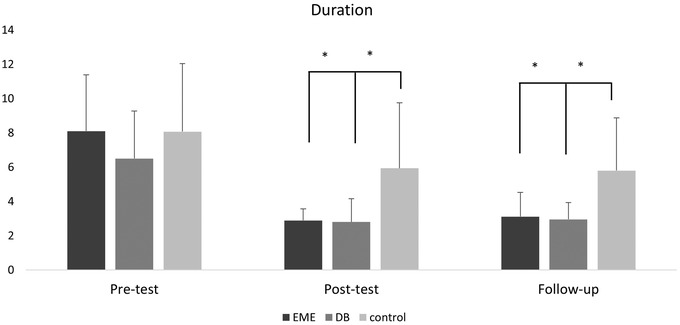
The duration included 4, 4–24, or 24–72 h per attack during the past 4 weeks—over 3 months. EME, eye movement exercise; DB, diaphragmatic breathing, and control across three assessment points—that is, pre‐test, post‐test, and follow‐up. Note. * = *p* < .05; ** = *p* < .001

The interaction effect of intervention and time was significant for the duration of migraine attacks (*F* (4, 100) = 4.362; *p* = .003). Likewise, the difference between the scores among the interventions also depends on the levels of the time factor. Significant main effects were also observed between the groups (*F* (2, 50) = 6.118; *p* = .004) and within the groups (*F* (2, 100) = 96.108; *p* = .0001). Therefore, regardless of each factor, there is a difference in duration between the levels of the other factor. Table 3 shows a pairwise comparison of the alpha‐adjusted simple effects of headache duration within groups. Furthermore, Figure 5 displays the pairwise comparison of simple effects between the groups for the duration of migraine attacks.

#### 3.1.3 Effects on intensity

**FIGURE 6:**
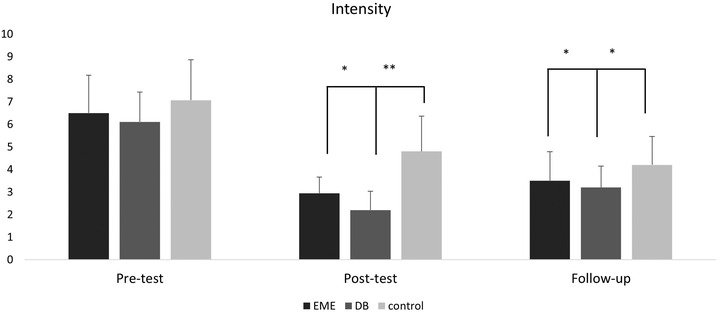
The intensity was moderate (1–3), severe (4–7), or worse case (8–10) per migraine attack during the past 4 weeks—over 3 months. EME, eye movement exercise; DB, diaphragmatic breathing, and control across three assessment points—that is, pre‐test, post‐test, and follow‐up. Note. * = *p* < .05; ** = *p* < .001

Like frequency and duration, in the intensity of migraine attacks, a significant interaction effect was observed between intervention and time (*F* (4, 100) = 3.780; *p* = 0.007). Even the main effect between the groups was significant for intensity (*F* (2, 50) = 13.588; *p* = .0001), and a significant difference was observed within the groups (*F* (2, 100) = 125.424; *p* = .0001). Pairwise comparisons of alpha‐adjusted simple effects of migraine intensity within the groups are shown in Table 3. The pairwise comparisons of the simple effects of migraine intensity between the groups are shown in Figure 6.

### 3.2 Secondary data analysis

The results of a series of Cochran's tests were used to assess changes in the individuals' menstrual cycle, frequency of OTC drug use, sleep pattern, and drinking water across the assessment points. They showed significant improvements for the interventional groups but not for the control group (Table 4). None of the participants in the interventional groups complained of adverse effects like muscle cramps, fatigue, chronic dizziness, or vomiting on exertion or after exercise.

**TABLE 4:** Results of Cochran's Q models testing inter‐group effects at post‐test and a 12‐month follow‐up

		Frequency					
Variables	Factors	Groups	Pre‐test	Post‐test	Follow‐up	Cochran's Q	p
Menstrual cycle	No effect: Increase	EME	13:5	18:0	18:0	10.00	.007
		DB	15:5	20:0	20:0	10.00	.007
		Control	10:5	11:4	14:1	6.50	.03
Over the counter	No use: Use	EME	0:18	2:16	4:14	4.80	.09
		DB	0:20	7:13	4:16	9.25	.01
		Control	2:13	1:14	1:14	0.66	.71
Wake up mode	Refreshed: Sleepy or tired	EME	5:13	18:0	18:0	26.00	.001
		DB	0:20	19:1	18:2	34.30	.001
		Control	3:12	1:14	0:15	4.66	.09
Sleep hours	Less than seven hours: More than seven hours	EME	16:2	9:9	12:6	5.69	.05
		DB	18:2	14:6	12:8	4.30	.11
		Control	13:2	13:2	13:2	0.00	1.00
Sleeping pattern	On time: Late	EME	4:14	12:6	15:3	14.92	.001
		DB	3:17	14:6	13:7	13.87	.001
		Control	2:13	1:14	1:14	0.66	.71
Drinking water	No: Yes	EME	14:4	1:17	0:18	24.40	.001
		DB	19:1	3:17	6:14	24.11	.001
		Control	14:1	13:2	13:2	0.66	.717

*Note*. No: the number of participants who did not use to drink water regularly at baseline, during the intervention (at post‐test), and during follow‐up; Yes: the number of participants who drank water daily at baseline, during the intervention (at post‐test), and during follow‐up. For example, at baseline (14:4), 14 participants did not drink water, and 4 regularly drank water. In the same group (i.e., EME), one participant did not drink water, while 17 did after the intervention (1:17). At the 12‐month follow‐up, all the participants were drinking water daily (0:18).

Abbreviations: EME, eye movement exercise; DB, diaphragmatic breathing.

Based on Table 4, migraine frequency decreased in all three groups during the menstrual cycle (*p* < .05). Therefore, regardless of the type of intervention (eye movement exercise, diaphragmatic breathing, or TAU), migraine attacks during menstruation have decreased. In interventional groups, the frequency of OTC drug use was significantly decreased (*p* < .05). Nonetheless, eye movement exercise and diaphragmatic breathing can improve and refresh the waking‐up mode and regulate sleep patterns (*p* < .05). Finally, the interventions have had some effects on the frequency of water drinking (*p* < .05).

Given the loss of participants from the post‐test to the follow‐up (see flowchart), we did not have a full compliance with the present trial intervention protocols. The compliance rates were: 53/63 × 100 = 84.1% compliance for the total sample; 20/22 × 100 = 90.9% compliance for the eye movement exercise group; 18/19 × 100 = 94.7% compliance for the diaphragmatic breathing; and 15/22 × 100 = 61.1% compliance for the control group.

## 4 DISCUSSION

The present trial assessed the effectiveness of eye movement exercise and diaphragmatic breathing with jogging in individuals with migraine. The first interventional group received eye movement exercise plus jogging, the second interventional group received diaphragmatic breathing plus jogging, and the control group only received TAU. The results showed significant effects of the interventions on migraine symptoms in the interventional groups (Figure 4 and Table 3). Therefore, practicing body–mind awareness and cognitive/network reconstruction‐based pieces of training (i.e., eye movement exercise and diaphragmatic breathing) in addition to regular aerobic exercises (i.e., jogging) can decrease the frequency, duration, and intensity of the attacks in a brain with migraine. The effectiveness could stem from various possible mechanisms, including: (a) brain–gut and neuroimmune axis (e.g., hypothalamus–pituitary–adrenal); (b) brain–brain reciprocal interlinks (e.g., hippocampus‐anterior cingulate cortex and medial prefrontal cortex oscillations); (c) circadian disruption and biomolecular dysregulation (e.g., oxidative stress and neurogenic inflammation); and (d) the body muscles, respiratory rate/depth, the flow of biomolecules/hormones (i.e., NO, BDNF), and adenosine monophosphate‐activated protein kinase (AMPK), each of which may play an initiating role in spurring migraine‐related symptoms (Fornari et al., 2020; Karapanou et al., 2009; Kempuraj et al., 2020; Ketchesin et al., 2020).

Although the results of a meta‐analysis (Naugle et al., 2012) suggested that the effect of aerobic exercise on individuals with migraine is only related to the frequency of the attacks, in a recent study (La Touche et al., 2020), aerobic exercises were assumed to influence all aspects of migraine symptoms, including frequency, duration, and intensity of the attacks. In the literature, the paradoxical findings about the effectiveness of aerobic exercises in individuals with migraines appear for the following reasons. First, based on the IHS recommendations (Silberstein et al., 2008; Tassorelli et al., 2018), one major drawback of earlier studies is heterogeneity in applying headache‐related outcome measures. Second, other studies have failed to address blinding, sample size, design, and randomization in their approach. Third, no study has compared the outcomes of aerobic exercises on individuals with migraine without aura and individuals with migraine with aura. Fourth, evidence was scarce for comparing the outcomes of aerobic exercises and pharmacological treatment of migraine symptoms. Fifth, no study on aerobic exercise has been complemented with other non‐medication/comfort therapies such as eye movement exercises or diaphragmatic breathing, or vice versa. Therefore, and based on findings from the brain with migraine studies, combining eye movement exercise or diaphragmatic breathing with jogging decreases kinesiophobia or sensitivity to movements in individuals with migraine (Goadsby et al., 2002; Rainero et al., 2020).

As a diagnostic criterion, individuals with migraine have a fear of movement or kinesiophobia (Benatto et al., 2019; Holmberg, 2020). To develop readiness for or potentiating neural responsiveness to a full‐body exercise and improve body awareness (Adler et al., 2014; Guyenet Patrice & Bayliss Douglas, 2015), the present trial provided individuals with eye movement exercise or diaphragmatic breathing (Murakami et al., 2014; Schurger et al., 2016, 2012). Considering neural or Hebbian learning, stimulating the brain on a regular basis adjusts brain functions at the molecular level (e.g., signaling mechanisms and memory formation) (Csermely et al., 2020). Eye movement exercises activate widespread, interconnected cortical and subcortical networks such as superior colliculus, oculomotor network in the brain stem, dorsolateral prefrontal brain cortex, basal ganglia, subthalamic structures, substantia nigra pars‐reticulata, visuomotor, parietal, and posterior cingulate cortices (Bolam et al., 2000; Burke et al., 2020; Rucker, 2010; Santamaria et al., 2020; Srivastava et al., 2018). Each network plays an excitatory or inhibitory role in pain processing (Caulo et al., 2019; Coiner et al., 2019). Therefore, performing eye movement exercises on a regular basis stimulates or potentiates all parts of the brain that are important in the experience of pain. The corneoretinal potential transduction by eye movement exercise is a bioelectrical signal, which is produced by two modes of polarities during eye movement exercise: a positively charged end (cornea) and a negatively charged end (retina) (Shepard et al., 2020). The conjugate eye movement exercise (i.e., from right to left or from top to bottom and vice versa) transduces sequential negative or positive electrical charges on the retina (Klein & Ettinger, 2019). These types of training‐induced transduction should potentiate synaptic plasticity and facilitate myelination mechanisms by: (a) regulating signal transmission (i.e., neuromodulation of ion channels and neurotransmitters), (b) synchronizing oscillations (i.e., frequency, amplitude, or phase), and (c) cortical reorganization or enhancement of timing within related cortical‐cortical or cortical‐subcortical brain networks (i.e., enhancement of memory, visuospatial accuracy, decision‐making, or task switching) (Hutton, 2008; Liu et al., 2015; Perrin et al., 1998; Pierrot‐Deseilligny et al., 2004; Slagter et al., 2017).

In the present trial, another essential guided behavior was diaphragmatic breathing. To be precise, jogging itself may increase exercise‐induced stress insults in a brain with migraine; however, diaphragmatic breathing helps promote stress‐response control indices and potentiate cortical readiness before jogging (Martarelli et al., 2011; Park et al., 2020). Diaphragmatic breathing can improve brain function via the following mechanisms: (a) chemical, which involves the regulation of biomolecules such as adenosine, melatonin, orexin, or calcitonin‐gene‐related peptide; (b) mechanical, which involves enhancing the rate, length, or intensity of breathing at the cortical level; and (c) cortical‐subcortical control, which is related to shared breathing centers and headache‐related networks such as the brainstem and medullary centers (Peña et al., 2020; Reiter et al., 2005; Sclocco et al., 2019; Vila‐Pueyo et al., 2018). Together, eye movement exercise plus jogging and diaphragmatic breathing plus jogging may induce mitochondrial oxidative phosphorylation, electrical transduction, and ionic and biomolecule homeostasis that can explain the primary and secondary outcomes of the present trial.

All participants in the present trial were female individuals with migraine. Because of the lack of generalizability of our findings to male individuals, a similar trial with the same approach and method should be performed with male individuals with migraine. Moreover, heterogeneity in the rate of prescribed medication and OTC drugs use in the control group was another limitation of the present trial. In the control group, we did not ask individuals to keep a journal of the medication that they were taking for their pain. However, no single or specific prescription has consistent or long‐term efficacy for migraine. Even recording the rate of medication use over two years by the control group individuals was considered an unviable goal and could not help interpreting the trial outcomes.

Furthermore, given the Middle Eastern context of the present trial, future studies can replicate the trial with individuals from different cultural, racial, and ethnic backgrounds. Since the acceptability and feasibility of eye movement exercise plus jogging or diaphragmatic breathing plus jogging intervention were not considered in the present preliminary trial, future studies are recommended to consider these criteria in their designs. Although the current comparative effectiveness trial was non‐inferiority (D'Agostino et al., 2003; Piaggio et al., 2006) and compared two interventions head‐to‐head with a third conventional intervention arm, the small sample size prevented us from supplying an inclusive conclusion. Future studies should replicate the present study's design with larger sample size. Future studies can also copy the outcomes of the present trial with other diseases and disorders like epilepsy, tinnitus, chronic dizziness, or vertigo (e.g., phobic postural vertigo, sleep apnea, or restless leg syndrome), and even for psychopathologies like depression and anxiety.

## 5 CONCLUSION

The primary outcomes of the present trial can be summarized as the positive effects of eye movement exercise plus jogging or diaphragmatic breathing plus jogging on decreasing the symptoms of migraine, including frequency, duration, and intensity of the attacks. Reduction in the menstrual cycle‐related headache attacks, use of OTC drugs, and improving sleep and drinking water are the outcomes that are secondary to primaries. Together, interventions used in the present trial offer promising prophylactic and therapeutic options for individuals with migraine.

## CONFLICT OF INTEREST

The authors do not have any conflict of interest.

## AUTHOR CONTRIBUTIONS

Mohammad Dawood Rahimi: conceptualization, method, data curation, investigation, project administration, and writing–original draft. Pouriya Hassani: investigation. Mohammad Taghi Kheirkhah: revision of formal analysis. Javad Salehi Fadardi: supervision, method, reviewing, and editing.

### PEER REVIEW

The peer review history for this article is available at https://publons.com/publon/10.1002/brb3.2820

## Funding information

It should be acknowledged that the present trial did not receive any full or partial financial support.

## Data Availability

The present manuscript includes all the generated or analyzed data. Datasets analyzed in the present trial are available from the corresponding author on reasonable request.
